# Retraction: Roles of Epigenetic Modifications in the Differentiation and Function of Pancreatic β-Cells

**DOI:** 10.3389/fcell.2022.885869

**Published:** 2022-03-18

The journal retracts the 28 August 2020 article cited above.

Following concerns regarding the originality of the article, an investigation was conducted in accordance with Frontiers’ policies. The investigation determined an unacceptably high level of similarity with an article published by Dai et al. (2020). This retraction was approved by the Field Chief Editor and the Specialty Chief Editor of *Frontiers in Cell and Developmental Biology*.

The authors concur with the retraction and sincerely regret any inconvenience this may have caused to the reviewers, editors, and readers of *Frontiers in Cell and Developmental Biology*.
